# Tetra-Dentate Cycloaddition Catalysts for Rapid Photopolymerization Reactions

**DOI:** 10.1021/acs.joc.2c02941

**Published:** 2023-04-08

**Authors:** Natanel Jarach, Hanna Dodiuk, Samuel Kenig, Shlomo Magdassi

**Affiliations:** † The Department of Polymer Materials Engineering, Pernick Faculty of Engineering, Shenkar College of Engineering Design and Art, Raman-Gan 5252626, Israel; ‡ Shlomo Magdassi Institute of Chemistry, Institute of Chemistry and the Center for Nanoscience and Nanotechnology, The Hebrew University of Jerusalem, Jerusalem 91904, Israel

## Abstract

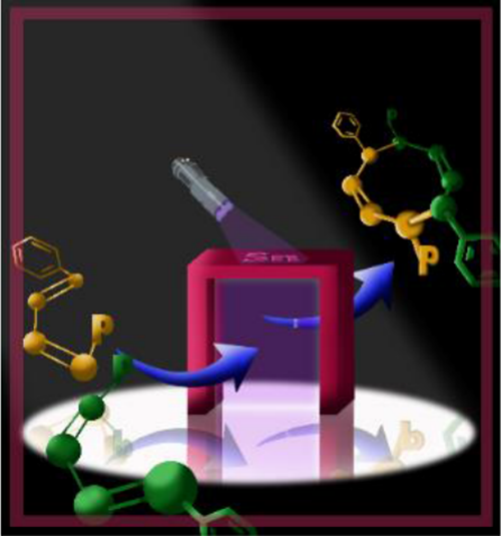

[4 + 4] and [2 + 2] cycloadditions are unique reactions since they form and deform cycloadducts under irradiation due to their inherent reversible nature. Whereas promising for the field of recycling, these reactions usually suffer from two major shortcomings: long reaction durations (hours) and the requirement of high-intensity light (∼100 W/cm^2^), typically at a short wavelength (<330 nm). We demonstrate several tetra-dentate catalysts that can overcome these fundamental limitations. Among them is a tin complex that enables 76% conversion within only 2 min of irradiation at 395 nm, much faster than the known ruthenium-based catalyst, under irradiation with light intensity two orders of magnitude lower than that reported in the literature. Due to the short photopolymerization time, low intensity (27 mW/cm^2^), and long UV light (395 nm), this unique complex opens new avenues for recycling three-dimensional printing products based on photopolymerization of cycloaddition reactions.

## 1 Introduction

Reversible covalent-bond-containing polymers (RCBPs), also known as vitrimers, covalent adaptable networks (CANs), or reorganizable polymers, are a new class of polymers that share a three-dimensional (3D) network-like structure with thermosets while having reprocessability and recyclability due to their dynamic or reversible cross-links.^1−4^ Among the reversible bonds studied in the literature, [2 + 2] and [4 + 4] cycloadditions are unique in having potential use in irradiation-based applications, such as photocuring of adhesives and stereolithography-based 3D printing. This potential results from the adduct formation under irradiation at specific wavelengths and dissociation under shorter ones.^1^ Unlike any other radiation-curing mechanisms, the complete dissociation of the adducts into their original components holds their potential for recyclability, followed by re-using in irradiation-based applications. Several such materials have been demonstrated in the literature. The most common ones are cinnamic acid and coumarin derivatives. Cinnamic acid derivatives undergo [2 + 2] cycloaddition to form cyclobutanes under irradiation at λ > 260 nm and undergo a reverse reaction under λ < 260 nm.^5−8^ Anthracene is the most common moiety to undergo [4 + 4] cycloaddition, which occurs under ∼350 nm irradiation. Whereas this is a longer wavelength than most [2 + 2]’s, it is still considered a harmful wavelength and shorter than that used in most photopolymerization-based applications and requires a relatively long reaction time for achieving complete conversion.^6,9−11^

Despite high potential in recycling, the [2 + 2] or [4 + 4] reaction faces two main obstacles preventing their use, especially in applications requiring short irradiation times, such as 3D printing and fast-cure adhesives: the reactions require irradiation at short wavelengths, and they are extremely slow (hours to days). Two main approaches were reported to overcome these shortcomings: catalyst (redshift and acceleration) and catalyst-free.

The catalyst-free approach, as was reported by Barner-Kowollik et al., introduces resonance-inducing groups, such as those found in styryl pyrene derivatives, into the reactive moieties. Introducing such groups reduces the band gap of the double bonds; thus, a shift of the operating wavelengths toward the visible-light spectrum is obtained.^12−17^ Nonetheless, sufficient cross-linking by light irradiation occurs only under long durations, at the time scale of hours.

Cycloaddition redshift catalysis is mainly based on photooxidation reagents, like ruthenium^18−22^ and iridium^23−28^ complexes and thioxanthone derivatives.^29−33^ Conductive^34^ and semiconductive^35^ particles or salts like pyrylium^36−38^ were reported for cycloreversion redshift. Two-photon beam can also be used as an alternative solution, as combining the two photons allows cycloaddition and cycloreversion to occur while being exposed to longer-light irradiation sources. Acceleration of [2 + 2] cycloaddition can be obtained using external factors, such as microwave irradiation,^39−41^ UV-flow reactors,^42,43^ or increased pressure during the reaction.^44^ The addition of accelerators, such as Lewis acid and base combinations or ion-containing solvents, was also found to be useful.^45−48^

To the best of our knowledge, the existing approaches still suffer from two main disadvantages: the reactions are too slow for fast-curing applications such as 3D printing, and they all require very high light intensity (up to 100 W/cm^2^ for >8 min^8,26^), which is much higher than that utilized in such applications. That being said, the applicability of cycloaddition under laser irradiation was demonstrated by Barner-Kowollik and co-workers^49^ who examined different [4 + 2] cycloaddition systems. These systems differ from the [2 + 2] and [4 + 4] cycloaddition reactions in which they exhibited spontaneous reversion in the absence of light and required irradiation at a time scale of hours.

Herein, we present tetra-dentate transition metals complexes catalysts that can initiate and accelerate cycloaddition reactions upon irradiation of light at the edge of the visible spectrum (λ > 385 nm) at very low intensity to achieve high conversion within very short irradiation durations. These catalysts, therefore, can be used to facilitate applications based on the reversible [4 + 4] and [2 + 2] cycloaddition bonds while requiring short photopolymerization times under irradiation with common low-intensity light-based printers.

## 2 Results and Discussion

**Figure 1:**
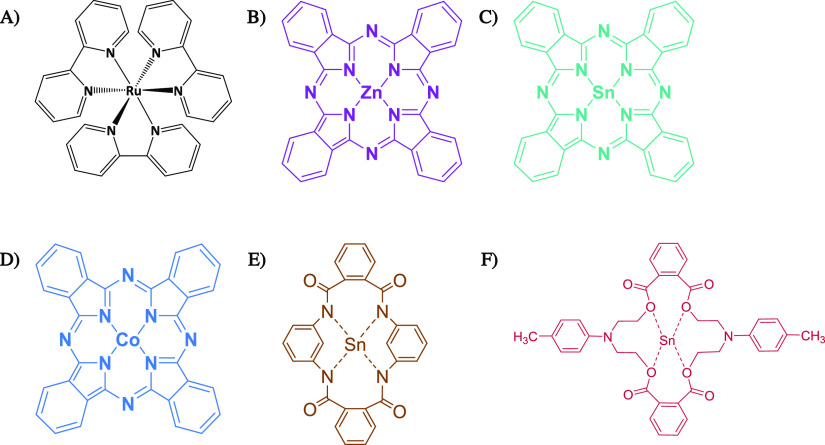
Schematic presentation of **Ru(bipy)_3_** (A), **ZnPC** (B), **SnPC** (C), **CoPC** (D), **Sn(MPDA-PA)** (E), and **Sn(PA-MPIB)** (F).

Ruthenium(*II*)tris(2,2′-bipyridine) [Ru(bipy)_3_] is the most common catalyst in the field of [2 + 2] and [4 + 4] cycloaddition reactions.^20,50−52^ However, this bidentate complex still suffers from the same disadvantages discussed above: high light intensity requirements and long reaction rates, unfitting for fast-curing applications such as 3D printing and fast-curing adhesives (e.g., dental adhesives). Herein, five tetradentate catalysts will be discussed as possible solutions to these challenges (Figure 1): zinc(*II*)phthalocyanine (ZnPC), tin(*II*)phthalocyanine (SnPC), cobalt(*II*)phthalocyanine (CoPC), tin(*II*) (2,2′-[(4-methylphenyl)imino]bisethylbisphthalate) (Sn(PA-MPIB)), and tin(II) (bis-N_1_,N_1_′-(1,3-phenylene)diphthalamide) (Sn(MPDA-PA)). The first two (Ru(bipy)_3_ and ZnPC) were commercially available and used without further processing. The other catalysts were synthesized, with the latter two being novel complexes (see Figure 1E,F).

The following results first compare three different core metals with the same ligand (phthalocyanine, which is known for its lack of toxicity and its light absorbance in the 360–405 nm range), and second, two additional tin-containing catalysts were compared to better understand the ligand effect on the catalysts’ reactivity. The chosen metals are cobalt, tin, and zinc, all transition metals with different electron densities and coordination abilities.

### 2.1 Catalyst Synthesis and Structural Analysis

The first phthalocyanine-containing complex, SnPC, was synthesized from SnCl_2_ and 1,2-dicyanobenzene in a microwave oven, resulting in a turquoise product that was ground to a powder and characterized using attenuated total reflectance infrared spectroscopy (ATR-IR), ^1^H NMR, and powder X-ray diffraction (XRD). Both ^1^H NMR spectra and XRD results (Figure S1-A) point to a complete conversion, with XRD showing that some of the catalysts were obtained in the form of SnPcCl_2_ (with two additional chlorides from the original SnCl_2_).^53^ It should be noted that as ATR-IR showed a major overlap between the different signals, it could not be used to identify the catalyst structure. CoPC was synthesized similarly to SnPC, with CoCl_2_ replacing SnCl_2_. All three characterization methods show similar results, with signal overlap in ATR-IR and a complete conversion based on ^1^H NMR and XRD results (Figure S2).

Sn(PA-MPIB) is a new complex synthesized in this study. The main difference between this complex and SnPC lies in the ligand: instead of phthalocyanine, a new ester containing tetra-dentate ligand is presented. This complex was synthesized in a solution [with ethanol (EtOH) as the solvent], reacting SnCl_2_, phthalic anhydride (PA), and 2,2′-[(4-methylphenyl)imino]bisethanol (MPIB), resulting in a crimson viscous liquid. Based on both ATR-IR and ^1^H NMR results, 86.23% conversion was calculated. As will be discussed later, this catalyst showed the highest efficiency (Figure 4): higher cycloaddition conversion at the short irradiation time: 76.0 ± 0.3% after 2 min.

Sn(MPDA-PA) is also novel, with an amide-containing tetra-dentate ligand with less flexibility and higher electron density. Sn(MPDA-PA) was synthesized in a solution (toluene as a solvent) from SnCl_2_, PA, and *m*-phenylenediamine (MPDA). The resulting product was dried overnight under vacuum at 70 °C and then ground into a brown powder, after which its structure was analyzed using ATR-IR, ^1^H NMR, and powder XRD. All three characterization methods showed 99.7% conversion.

**Figure 2:**
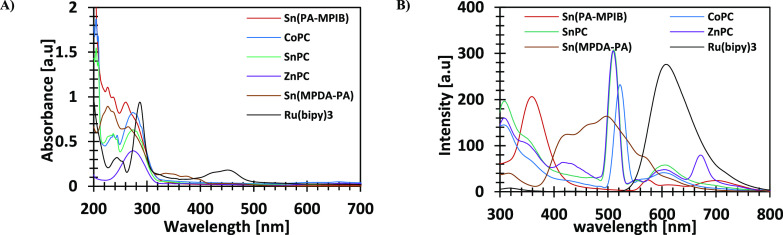
Absorbance (A) and fluorescence (B) of **Sn(PA-MPIB)** (crimson), **CoPC** (blue), **SnPC** (turquoise), **ZnPC** (purple), **Sn(MPDA-PA)** (brown), and **Ru(bipy)_3_** (black). The tests were conducted using ethanol (EtOH) as a solvent with 1.25 × 10^–11^ [M] and 1.81 × 10^–2^ [M] for absorbance and fluorescence, respectively.

The absorbance of all six catalysts was measured to understand their reactivity under irradiation. As shown in Figure 2A, up to ∼390 nm, the highest absorbance in the range of 360–405 nm was obtained by Ru(bipy)_3_, despite not being the most efficient catalyst. The fluorescence of the complexes was also measured using the same conditions under excitation of 255 nm.

### 2.2 Prepolymer Synthesis and Structural Analysis

**Figure 3:**
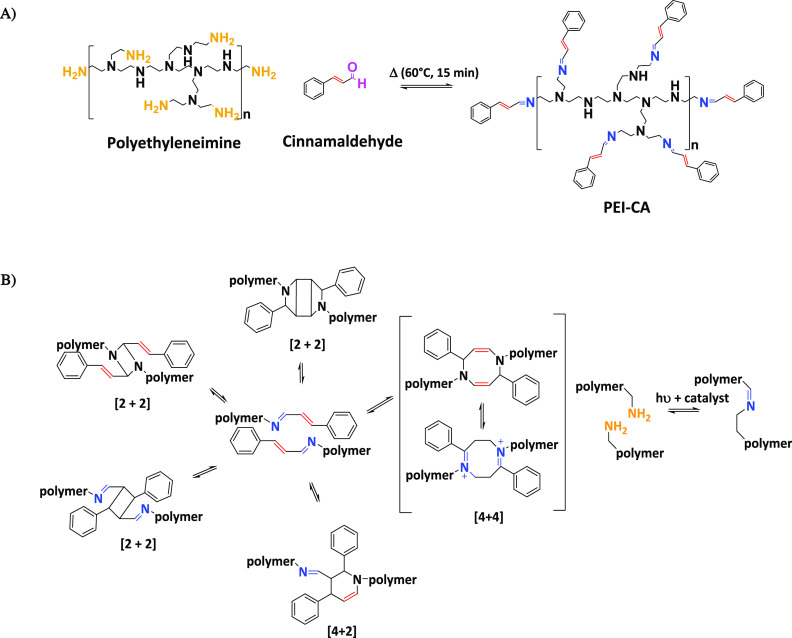
Schematic illustration of PEI-CA prepolymer synthesis from CA and PEI (A) and the different possible curing reactions (B).

As a model for RCBPs, a prepolymer with the potential to undergo [2 + 2] or [4 + 4] cycloaddition^5−8^ was synthesized from polyethyleneimine (PEI) and cinnamaldehyde (CA) (Figure 3A). In the synthesis, it was found that adding the tetradentate catalysts caused an acceleration in the imine formation, shortening the reaction from half an hour to 2 min only. It should be noted that the light intensity used to cure this polymer is extremely low compared to that reported in the literature, 27 mW/cm^2^ and ∼100 W/cm^2^,^8,26^ respectively. The chemical composition of **PEI-CA** was identified using ATR-IR, ^1^H NMR, UV–vis, and fluorescence. IR spectra (Figure S5-A) of the polymer showed the formation of imine groups (∼1630 cm^–1^^54,55^) and the disappearance of aldehyde groups (∼1700 cm^–1^^54,56^). ^1^H NMR also demonstrated this phenomenon (following the changes in the signals at 3.34–3.5, ∼8, and ∼7 ppm^57^). Based on these results, an 80.3% conversion was achieved.

**Figure 4:**
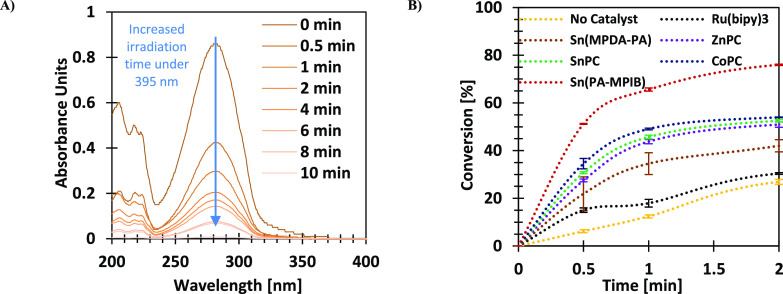
Example of the changes of the absorbance spectra of PEI-CA with **Sn(PA-MPIB)** as a catalyst (A) and conversion up to 2 min after irradiation under 395 nm lamp (27 mW/cm^2^) using different catalysts (B).

The main problem of the latter pre-polymer is its potential to undergo several different reactions: [2 + 2] cycloaddition, [4 + 4] cycloaddition, [4 + 2] cycloaddition, and even amine oxidation into imines after long irradiation time^58,59^ (see Figure 3B). As these products have similarities with the original pre-polymer, it was difficult to determine the curing conversion based on “classical” IR and/or NMR methods. Thus, following an alternative method suggested by Rahimi et al. and Yano et al.,^60,61^ the conversion was calculated based on the changes in the absorbance signal at 280–290 nm (see example in Figure 4A) and the correlative fluorescence signal at 320–350 nm. Since the formation of the cycloadducts occurred only after irradiation and [4 + 2] is a forbidden transformation under these conditions (it might occur while heating the polymer),^62^ it can be concluded that most of the products obtained were due to [2 + 2] or [4 + 4] cycloaddition reactions. The evaluation of **PEI-CA** conversion was based on the minimal concentration of the catalyst that results in the formation of an insoluble polymer after ∼10 min irradiation. 1 mol % of the catalysts was found to be the minimal concentration for all but Sn(PA-MPIB), for which it was 0.75 mol %. The conversion over time following irradiation under 395 nm was evaluated and compared with the conversion of **PEI-CA** without a catalyst. As shown in Figures 4B and S6, Sn(PA-MPIB) was the most efficient, even at a reduced concentration (0.75 mol % compared to 1 mol % of the others). Moreover, all the tetra-dentate catalysts were found to be more efficient than the common Ru(bipy)_3_ used as the reference in this study, despite the use of two orders of magnitude lower intensity than the ruthenium literature-reported systems.

In some photolithography-based processes, the polymer is only required in the first stage to reach such a conversion that the structure can be preserved. Then, only in the second stage, known as the post-curing, the irradiation continues, and the material reaches its full conversion. For most polymers, ∼60% conversion can be considered high enough for the first stage; thus, when using Sn(PA-MPIB) in 3D printing, 40 s may be enough.

### 2.3 Mechanism and Catalysts’ Efficiency

A thorough comprehension of the photocuring mechanism is necessary to understand the differences in the catalysts’ performance. During a direct excitation of [2 + 2] and [4 + 4] cycloadditions, a double-bond containing moiety goes through transformation between the ground state (S_0_) and the singlet state (S_1_), following irradiation. Then, the excited moiety reacts with another double bond-containing moiety to form the cycloadduct. In most cases, nonconjugated alkenes tend to have a high energy level S_1_, which requires short wavelengths irradiation (like in the case of CA derivatives, which require ∼260 nm). Moreover, since the S_1_ state is short-lived, it results in internal conversion and fluorescence effects that compete with the cycloaddition reaction.^63^

Electrons can populate S_1_ both by direct excitation and by energy transfer from another photoexcited molecule (known as the sensitizer, which, in this study, is the catalyst). Once the catalyst is in its S_1_ state, it transfers its energy to the double bond of the desired reagent. Using this mechanism, much longer wavelengths can be used compared to direct excitation. Another path is photoinduced electron transfer (PET), which occurs not by the reagent excitation but by the catalyst oxidation or reduction. First, an intermediate radical anion or cation on the double bond of the reagent is formed through the photochemical pathway. Then, by reacting two intermediate moieties, cyclobutane forms.^63,64^ As will be discussed later, some of the catalysts (like ruthenium, after long irradiation time) operate using the PET mechanism, while others might function via the first mechanism.

Analysis of the reactions’ mechanism indicates that the catalysts’ efficiency (the conversion at short irradiation time) differences stem from two main factors: electron transfer efficiency between the catalyst and the polymer and the favorable curing reaction: [2 + 2] or [4 + 4]. The electron transfer efficiency may result from a better coordination ability or a good matching between the two components’ orbitals. Electron counting could help understand coordination abilities. Ru(bipy)_3_ is an 18e̅ complex with a short Ru–N distance (∼2.05 Å);^65^ thus, its ability to coordinate other ligands, in this case, the double bonds of **PEI-CA**, is limited. ZnPC is also an 18e̅ complex, while SnPC is a 20e̅ complex, but since Sn and Zn complexes can violate the “18 electrons law”, coordination between Z/SnPC and **PEI-CA** is still possible. Moreover, SnPC can coordinate up to eight more electrons compared to ZnPC.^66^ However, some of it was obtained in the form of SnPCCl_2_, limiting its efficiency (both sterically and by occupation of orbitals); thus, no significant differences between the two were observed.

CoPC is a 15e̅ complex; thus, it can still coordinate with some double bonds of **PEI-CA**, probably with a little more efficiency than Z/SnPC due to lower electron density. Sn(PA-MPIB) is, like SnPC, a 20*e̅* complex, and similarly can still coordinate with **PEI-CA** as the core metal is the same. Furthermore, unlike Co, the Sn complex can coordinate two double bonds on the same side of the complex.^66^ As the efficiency of the cycloaddition depends on both the electron transfer from the catalyst and the efficient overlap between two double-bond orbitals, the fact that the two double bonds coordinate in an optimal steric arrangement in correlation to the tetra-dentate ligand makes it more efficient and accelerates the reaction. Moreover, the ligands themselves are different: phthalocyanine is a planar aromatic ligand while the PA-MPIB ligand is nonplanar, and the existence of ethyl segments makes it more flexible. This flexibility can lead to geometric changes in the ligand and contribute to a higher degree of coordination of the double bonds of **PEI-CA**.

In Sn(MPDA-PA), replacing the esters with amides made the ligand a better electron-donating component, making Sn electron-richer; thus, its ability to coordinate with **PEI-CA** is reduced. Moreover, MPDA-PA is stiffer than PA-MPIB, lacking the ethyl segments and its nitrogens are electron richer than phthalocyanine’s; thus, Sn(MPDA-PA) is less effective than ZnPC, SnPC, and CoPC. The differences between the coordination abilities might also be observed in the fluorescence spectra (Figure S6). More efficient coordination leads to a more efficient electron transfer from the catalyst to the polymer. Efficient electron transfer will result in fewer emission signals as the electrons are transferred to the polymer instead of relaxing into the ground state. Indeed, whereas only two emission signals were observed for both Sn(PA-MPIB) and CoPC (the most efficient catalysts), the others have three or more signals.

**Figure 5:**
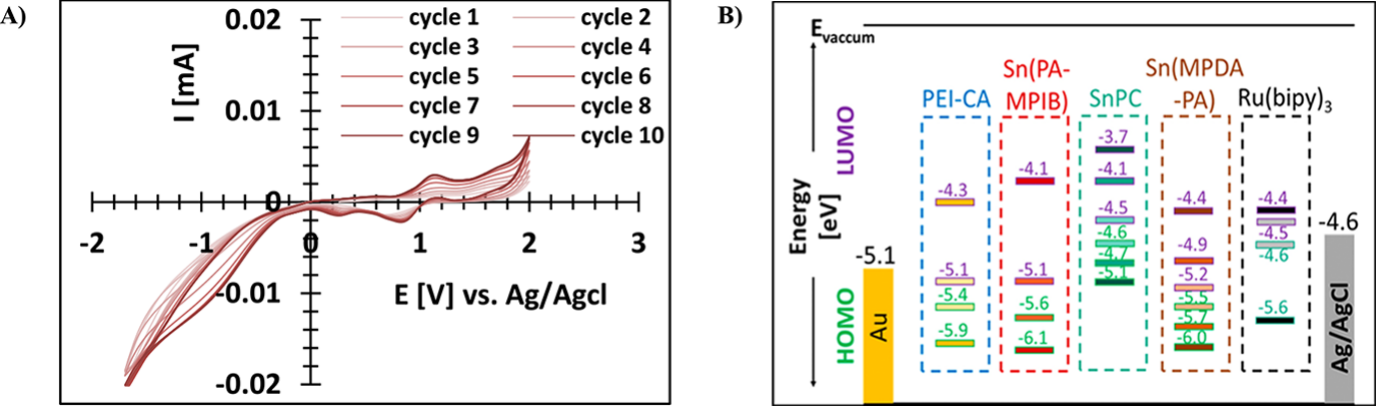
(A) Example of CV plot of 10 cycles of **Sn(MPDA-PA)** (see also Figure S8). All CV measurements were conducted in 0.2 M CHCl_3_ solution with 0.2 M TBABF_4_. The used electrodes were gold (working electrode, −5.1 eV), Ag/AgCl (reference electrode, −4.6 eV), and platinum (counter electrode). (B) Molecular orbitals energies (HOMO/LUMO) of **PEI-CA** (blue), **Sn(PA-MPIB)** (crimson), **SnPC** (turquoise), **Sn(MPDA-PA)** (brown), and **Ru(bipy)_3_** (black) as was measured by CV. HOMO and LUMO levels labeled with different colors: HOMO – green and LUMO – purple. The estimation of HOMO and LUMO levels was according to oxidation and reduction offsets.

The electron transfer efficiency is mainly derived from good fitting between two-component orbitals. Thus, the highest occupied molecular orbital (HOMO) and the lowest unoccupied molecular orbital (LUMO) energy level values can be used to compare the different components’ efficiency. The determination was done by cyclic voltammetry (CV) measurements, as typically shown in Figure 5A. Hence, the three Sn catalysts and Ru(bipy)_3_ were investigated and compared with **PEI-CA**. As shown in Figure 5B, the LUMO level of **PEI-CA** lies between Sn(PA-MPIB)’s highest measured orbital and the second LUMO. Thus, when exciting an electron up to the highest level, it is more likely that it will relax into the LUMO of **PEI-CA** rather than into the much lower energy level of the Sn(PA-MPIB)’s orbital. SnPC, however, has two orbitals above those of **PEI-CA**; thus, the excited electron is first relaxed to the second LUMO and only then transfer into the close **PEI-CA** LUMO. Electron donation properties of the MPDA-PA ligand resulted in Sn(MPDA-PA)’s orbitals being on a lower energy level than those of **PEI-CA**, much like Ru(bipy)_3_. Therefore, although electrons can transfer, the process will be less efficient.

Additional explanation for the Sn(PA-MPIB) efficiency can be supported by the redshift of the absorbance and fluorescence of **PEI-CA**. With no catalyst, **PEI-CA** UV–vis λ_max_ = 280 nm, whereas when using Ru(bipy)_3_ and Sn(MPDA-PA), it is λ_max_ = 285 nm, for CoPC, it is λ_max_ = 290 nm and for Sn(PA-MPIB), it is λ_max_ = 293 nm. The fluorescence also shows changes: **PEI-CA** λ_max_ = 320 nm, Ru(bipy)_3_ λ_max_ = 380 nm, CoPC λ_max_ = 390 nm, and Sn(PA-MPIB) λ_max_ = 396 nm. These results might point to a reduction in the **PEI-CA** optical band gap in the presence of the catalysts, with the highest decrease with Sn(PA-MPIB). As lower gap results in a more efficient reaction, Sn(PA-MPIB) causes the highest acceleration, probably due to its better coordination with the polymer (due to more available bonding sites).

**Figure 6:**
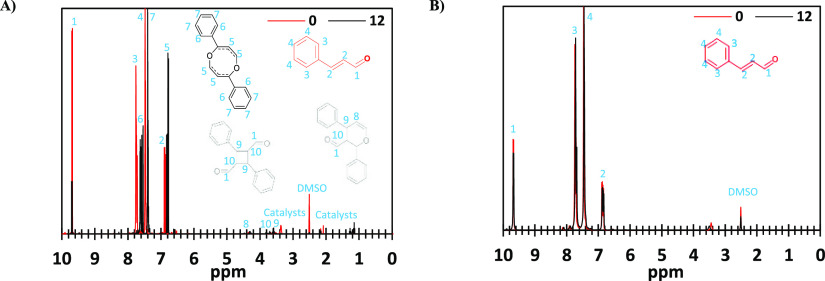
^1^H NMR (400 MHz, CDCl_3_) comparison of the CA dimerization process using **Sn(PA-MPIB)** (A) and **CoPC** (B) before irradiation (red) and after 12 min (black). The dimerization was obtained after irradiation for 12 min, under 395 nm lamp (27 mW/cm^2^).

Favoring [4 + 4] over [2 + 2] cycloaddition may also affect the catalysts’ efficiency, as the [4 + 4] adduct is more thermodynamically stable. To prove this assumption, CoPC, Sn(PA-MPIB), and Sn(MPDA-PA) were used to initiate CA-dimerization, using the same concentrations (1, 0.75, and 1 mol %, respectively). These catalysts were chosen as the least and the two most efficient (the lowest and highest conversion after 2 min) of the tetra-dentate complexes. As shown in Figures 6A,B and S7, after 12 min, only Sn(PA-MPIB) caused dimerization, with [4 + 4] being the dominant product. This can enable understanding the differences between the catalysts. Sn(PA-MPIB) forms preferentially [4 + 4] cycloadducts probably because it can coordinate more than one double bond on the same side of the tetradentate ligand. [4 + 4] cycloadditions, besides forming a more stable adduct, are probably faster than [2 + 2], and thus, Sn(PA-MPIB) accelerates curing more than CoPC, whose ability to form [4 + 4] cycloadducts is lower. Sn(MPDA-PA) is more electron-rich than both CoPC and Sn(PA-MPIB) and thus less effective in this case. This may also explain the changes in the conversion trends of Ru(bipy)_3_ and **PEI-CA**; up to 4 min, only [4 + 4] occurs, which is less efficient for these two. After that time, [2 + 2] starts; thus, the conversion trend changes.

The major differences between CA-dimerization and **PEI-CA** curing are the differences between imine and aldehydes and the geometrical proximity of the functional groups. These differences probably result in CoPC being more efficient for polymer curing than CA dimerization.

## 3 Conclusions

[4 + 4] and [2 + 2] cycloaddition reactions are limited due to the current requirement of high light intensity, short wavelength UV, and long reaction times. Thus, there is a profound need for efficient catalysts for cycloaddition reactions that can remedy these shortcomings. Herein, five complexes were evaluated as catalysts for the cycloaddition reactions. All five achieved higher cycloaddition conversion at shorter irradiation times than the common ruthenium catalyst. The newly synthesized Sn(PA-MPIB) complex was the most efficient, displaying more than the double conversion level than the ruthenium, reaching 76.0 ± 0.3% conversion within 2 min only. This conversion was achieved under irradiation intensity lower by two orders of magnitude than that reported for the ruthenium complex. Specifically, irradiation for 2 min at a low intensity of 27 mW/cm^2^ was used for the new complex, compared to more than 8 min at a much higher intensity of ∼100 W/cm^2^ for the ruthenium complex.

Catalyst performance was affected by the electron transfer efficiency of the catalyst polymer and the preference for the [4 + 4] cycloaddition reaction over the [2 + 2] reaction. It was concluded that the “optimized catalyst” should be based on a poor-electron complex with a transition metal core that can violate the “18 electrons rule”, containing a tetra-dentate ligand with a preference for (semi-)flexible ligands. This new catalyst opens the path to advance RCBPs into industrial applications that require fast photocuring, such as 3D printing and advanced adhesives. Furthermore, the new catalyst will enable the synthesis of polymeric materials with reversible covalent bonds, which is important for recycling of radiation-cured polymers.

## 4 Methods

### 4.1 Materials

Polyethyleneimine (PEI, branched, Mw 800), 1,2-dicyanobenzene, tin(*II*) chloride (SnCl_2_), cobalt(*II*) chloride (CoCl_2_), zinc phthalocyanine (ZnPC, 96%), phthalic anhydride, 2,2′-[(4-methylphenyl)imino]bisethanol, and *m*-phenylenediamine were all supplied by Sigma-Aldrich, Israel. *Trans*-cinnamaldehyde (CA, 99%) was supplied by Rhenium, Israel. Ethanol (EtOH) 96% was supplied by Biolab-Chemicals Inc., Israel. All materials were used without further treatment.

### 4.2 Catalysts Synthesis

For more details on the characterization instruments, see Section 4.5.

Based on previous studies,^67^ tin(*II*) phthalocyanine (SnPC) and cobalt(*II*) phthalocyanine (CoPC) were synthesized using a microwave oven (MW2031W, Sauter, Groupe Brandt, France) in an open vessel. Tin(*II*) chloride (SnCl_2_) or cobalt(*II*) chloride (CoCl_2_) and 1,2-dicyanobenzene were reacted in a 1:4 molar ratio (metal salt: 1,2-dicyanobenzene). At first, the components were dry-blended until a homogenous powder mixture was obtained. Then, the mixture was heated under 500 W microwave irradiation for 2 min. To overcome the high exotherm, the microwave was stopped for 15 s every 30 s. SnPC analysis: ^1^H NMR (500 MHz, DMSO-d6): δ 8.15 (m, *J* = 5.7 Hz, 8H), 7.94 (m, *J* = 5.7 Hz, 8H). UV–vis (absorbance): λ_max_ = 665, 275, 237, 205 nm. CoPC analysis: ^1^H NMR (400 MHz, DMSO-d6): δ 8.13 (s, 8H), 7.92 (s, 8H). UV–vis (absorbance): λ_max_ = 665, 274, 238, 205 nm.

Tin(*II*) (2,2′-[(4-methylphenyl)imino]bisethylbisphthalate) (Sn(PA-MPIB)) was synthesized in a solution as follows: 2 g of SnCl_2_, 3.12 g of PA, and 3.53 g of MPIB (1:2:2 molar ratio) were dissolved in 50 mL of acetone using ultrasonication bath at room temperature. Then, the solution was heated at 70 °C (in a silicon oil bath) and stirred with a magnetic stirrer. A few droplets of HCl (37%) solution were added to obtain a pH of 6. Immediately after HCl addition, the solution color changed from pale white to red and then deep red. After an additional hour, the temperature was increased to 120 °C, followed by the addition of 50 mL of ethanol (EtOH, 100 mL solvents overall). After an hour, the reflux was stopped, and ethanol was evaporated under heating at the same temperature to give a crimson high viscous liquid. The liquid was heated at 140 °C for one more hour and then was cooled to room temperature, obtaining a dark crimson highly viscous liquid. Analysis of the complex was as follows: IR (ATR-IR): 1698 cm^–1^. ^1^H NMR (400 MHz, DMSO-d6): δ 7.74 (d, *J* = 8.4 Hz, 4H), 7.59 (d, *J* = 6.96 Hz, 4H), 7.24 (q, *J* = 8.5, 4H), 7.00 (d, *J* = 8.4 Hz, 4H), 4.21 (q, *J* = 5.8 Hz, 8H), 3.49 (m, 8H), 2.66 (t, *J* = 7.0, 6H). As shown in Figure S3-B, the residential reagents (mostly MPIB) can be seen at: 1.5 ppm, 3.18 ppm, 3.74 ppm, and 4.37 ppm. UV–vis (absorbance): λ_max_ = 263, 227, 200 nm. When comparing C=O anhydride IR signals (∼1840 cm^-1^^54,55^) normalized to phthalic anhydride (PA) aromatic C–H signals (∼706 cm^-1^,^54,55^ which are a somewhat different form the 2,2′-[(4-methylphenyl)imino]bisethanol [MPIB] aromatic signals), a 86.23% conversion can be calculated. The same conversion can also be calculated when following ^1^H NMR (Figure S3-B), either by monitoring the addition of CH_2_–O–CO (4.21 ppm^57^) or the disappearance of MPIB CH_2_–OH (3.99 ppm^57^). Moreover, as shown in Figure S3-B, the residual reagents (mostly MPIB) can be seen at 1.5, 3.18, 3.74, and 4.37 ppm.

Tin(*II*) (bis-N_1_,N_1_’-(1,3-phenylene)diphthalamide) (Sn(MPDA-PA)) was also synthesized in a solution. SnCl_2_ (2 g), 3.12 g of PA, and 2.3 g of MPDA (1:2:2 molar ratio) were dissolved in 200 mL of toluene using an ultrasonication bath. The solution was stirred with a magnetic stirrer and heated at 140 °C for three hours (in a silicon oil bath). A few droplets of HCl (37%) solution were added to obtain a pH 6, followed by an immediate color change to dark crimson. A brown solid was obtained after completely drying under a vacuum oven at 70 °C overnight and grinding into powder. The complex structure was analyzed as follows: IR (ATR-IR): 1770 cm^–1^, 1712 cm^–1^. ^1^H NMR (400 MHz, DMSO-d6): δ (ppm) 7.99 (m, 8H), 7.92 (m, 2H), 7.71 (t, *J* = 2.4 Hz, 2H), 7.58 (m, 4H). UV–vis (absorbance): λ_max_ = 480, 335, 267, 228 nm. All three characterization methods (ATR-IR, ^1^H NMR, and XRD) showed that 99.7% conversion was obtained: following the disappearance of anhydride C=O signals (∼1840 cm^-1^^54,55^) normalized to PA aromatic C–H signals (∼706 cm^–1^ 125,126) in the ATR-IR results (Figure S4-A), no *m*-phenylenediamine (MPDA) NH_2_ signals (6–7 ppm^57^) were observed on the ^1^H NMR results (Figure S4-B). Furthermore, no evidence of the reagent crystallinity in the XRD results (Figure S4-C) was detected. The XRD results highlight the novelty of the catalyst as no similar ligand XRD was previously reported in the literature.

### 4.3 Prepolymer (PEI-CA) Synthesis

The pre-polymer (**PAI-CA**) was synthesized following a known aldehyde and polyethyleneimine (PEI) reaction.^3,68^ Following their equivalent weight calculation and the fact that PEI consists of 25% primary amines, 50% secondary amines, and 25% tertiary amines, PEI was reacted with cinnamaldehyde (CA) in a molar ratio of 1:15.71 (PEI–CA). Different catalysts’ ratios were tested: from 0.5 mol % (of the total reagents) to 1 mol %. At first, the chosen catalyst was dissolved and mixed in CA using 15 min ultrasonication bath (15 Hz, Elmasonic P, Elma Schmidbauer GmbH, Germany) at 60 °C and vortex mixing until reaching a homogenous mixture. The mixture was then added to a pre-heated 60 °C (in a silicon oil bath) PEI during mixing. After 5 min at 60 °C, the mixture was put under 15 min ultrasonication at 60 °C. Structural analysis was as follows: IR (ATR-IR): 3100, 1707, 1674, 1630 cm^–1^. ^1^H NMR (500 MHz, CDCl_3_): δ (ppm) 7.98, 7.55, 7.31, 6.87, 6.52, 3.59, 3.00, 2.56, 1.27. All broad peaks are due to polymeric molecular weight distribution. UV–vis: λ_max_ = 280 nm. Fluorescence (excitation: 280 nm): λ_max_ = 320 nm. Comparing the ^1^H NMR aldehyde signal (9.7 ppm,^57^ labeled 5 in Figure S5) disappearance normalized to aromatic C-H signals (7.7 ppm,^57^ labeled 6 in Figure S5) demonstrated 80.3% conversion.

### 4.4 Curing

The pre-polymer (PEI-CA) was cured under a 395 nm lamp (27 mW/cm^2^, 244.90 Hz, Radiant Flux = 1.12 W, Quantum Flux = 892 [photons/(m^2^s)], Integration Technology Ltd., UK. Figure S9) for different time periods: 0.5, 1, 2, 4, 6, 8, 10, 12, and 14 min.

### 4.5 Structure, Conversion, and Curing Analysis

To analyze prepolymer’s, cured polymer’s, and catalysts’ compositions, IR spectroscopy, NMR, and X-ray diffraction (XRD) were used. IR was recorded using the ATR-IR method on a Bruker Alpha-P machine (Brucker, USA), in the range of 400–4000 cm^–1^. ^1^H NMR was tested using CDCl_3_ or DMSO-6D as a solvent and was performed in a 500 and 400 MHz spectrometer (Ascend 500 Neo and Ascend 400 Neo by Brucker, USA) with tetramethylsilane (TMS) as an internal reference. XRD results were recorded using a thin-film powder diffraction instrument (λ_Cu_ K_α_ = 1.5406 Å, Shimadzu XRD-6000, Shimadzu, Japan). Absorbance spectra of catalysts were recorded using a UV–Vis–NIR spectrophotometer (UV-1800, Shimadzu, Japan) between 800 and 200 nm with 1.25·10^–11^ [M] concentration in ethanol (EtOH) in 1 cm path length quartz cuvettes. Their fluorescence was recorded (Cary Eclipse Fluorescence Spectrometer, Agilent, US) in 1 cm path length quartz cuvettes between 300 and 600 nm, using EtOH as a solvent, following excitation of 285 nm and using EtOH solution (6.9 mg/mL [gr/ml]).

Curing conversion was analyzed using ultraviolet–visible (UV–Vis) absorbance and fluorescence tests of dissolved 1 cm diameter and 1 mm thickness cured discs. The samples were tested in EtOH solution (2.81 × 10^–2^ [mg/ml]) with 1 cm path length quartz cuvettes. The same method was also used to measure cinnamaldehyde dimerization under the same irradiation conditions, although the concentration of cinnamaldehyde in EtOH for UV–Vis’ spectra was 1.91 × 10^–6^ [M].

Polymers’ conversion was measured following the changes of absorbance in 280–288 nm, a known signal of unsaturated aldehyde and/or imine,^68,69^ which may be found in cinnamaldehyde or the prepolymer, respectively. Polymer conversion was also measured following the changes of these signals’ fluorescence. As was discussed, only neglectable changes were found between the two methods. The conversion was calculated as follows (eq 1) by assuming that the width at half-height is equivalent, where Int_0_ refers to intensity before irradiation and *Int_t_* refers to intensity at a specific irradiation time:
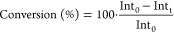
1

### 4.6 Energy Level Calculations

Molecular orbitals energies (HOMO/LUMO) of **PEI-CA**, Sn(PA-MPIB), SnPC, Sn(MPDA-PA), and Ru(bipy)_3_ were measured by cyclic voltammetry (CV. VSP, BioLogic Science Instruments, France). The test was conducted in 0.2 M CHCl_3_ solution with an addition of 0.2 M tetrabutylammonium tetrafluoroborate (TBABF_4_). The used electrodes were gold (working electrode, −5.1 eV), Ag/AgCl (reference electrode, −4.6 eV), and platinum (counter electrode) with . The estimation of HOMO and LUMO levels versus vacuum was according to oxidation and reduction offsets, following eqs 2 and 3, respectively.
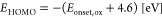
2
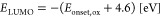
3

## Data Availability

The data underlying this study are available in the published article and its Supporting Information.

## Supporting Information Available

SnPC structural analysis; CoPC structural analysis; Sn(PA-MPIB) structural analysis; Sn(MPDA-PA) structural analysis; PEI-CA structural analysis; a full UV–vis absorbance spectra and cycloaddition conversion for all compositions; UV–vis absorbance for dimerization analysis; all cyclic voltammetry results; and emission spectrum of the light source (PDF)
