# Supplementary material for: Tetra-Dentate Cycloaddition Catalysts for Rapid Photopolymerization Reactions
Source: J Org Chem. 2023 Apr 8;88(9):5359–67. doi: 10.1021/acs.joc.2c02941 (PMC10167662; doi:10.1021/acs.joc.2c02941)
Supplement: Supplementary file 1 — jo2c02941_si_001.pdf [file jo2c02941_si_001.pdf]

Supplementary Information

Tetra-Dentate Cycloaddition Catalysts for Rapid Photopolymerization Reactions

Natanel Jarach [a,b], Hanna Dodiuk[a], Samuel Kenig[a], Shlomo Magdassi*[b]*

[a] Natanel Jarach, Hanna Dodiuk, and Samuel Kenig. The Department of Polymer Materials Engineering, Pernick Faculty of Engineering, Shenkar – Engineering. Design. Art, Raman-Gan, , 5252626, Israel. E-mail: hannad@shenkar.ac.il

[b] Natanel Jarach, Shlomo Magdassi Institute of Chemistry, Institute of Chemistry and the Center for Nanoscience and Nanotechnology, The Hebrew University of Jerusalem, Jerusalem, 91904, Israel. Email: magdassi@mail.huji.ac.il

**Corresponding authors:*

Hanna Dodiuk – E-mail: hannad@shenkar.ac.il

Shlomo Magdassi – Email: magdassi@mail.huji.ac.il

CONTENTS

Fig. S1 . **SnPC Analysis.** ^{1}H-NMR (500 Hz) in DMSO-6D of **SnPC** (**A**), powder XRD of the complex (**B**) and an interpretation of the signals (**C**). S4

Fig. S2 . **CoPC Analysis.** ^{1}H-NMR (400 Hz) in DMSO-6D of **CoPC** (**A**), powder XRD of the complex (**B**) and an interpretation of the signals (**C**). S6

Fig. S3. **Sn(PA-MPIB) Analysis.** ATR-IR of PA (blue), MPIB (pale orange), and Sn(PA-MPIB) (black) (**A**) and ^{1}H-NMR (400 Hz) of the complex in DMSO-6D (**B**). S7

Fig. S4. **Sn(MPDA-PA) Analysis.** ATR-IR of PA (blue), MPDA (pale orange), and Sn(MPDA-PA) (black) (**A**) and ^{1}H-NMR (400 Hz, DMSO-6D) (**B**) and XRD (**C**) of the complex. S9

Fig. S5. **PEI-CA Analysis.** A schematic illustration of PEI-CA pre-polymer synthesis from CA and PEI (**A**) and their structure analysis by ATR-IR (**B**) and ^{1}H-NMR (500 Hz) in $CDCl_3$ (**C**). For both: CA (yellow), PEI (orange) and the pre-polymer **PEI-CA** (black). S10

Fig. S6. PEI-CA absorbance's and fluorescence (285 nm excitation) changes after irradiation under 395 nm lamp (27 W\cm^2): without a catalyst (**A, B**), and with $Ru(bipy)_3$ (**C, D**), Sn(MPDA-PA) (**E,F**), ZnPC (**G,H**), SnPC (**I,J**), CoPC (**K,L**), and Sn(PA-MPIB) (**M,N**). The curing conversion (%) as a function of irradiation time (min) of the pre-polymer with and without the tested catalysts (**O**), where: **PEI-CA** neat (yellow), **Ru(ipy)$_3$** (black), **Sn(MPDA-PA**) (brown), **ZnPC** (purple), **SnPC** (turquoise), **CoPC** (blue), and **Sn(PA-MPIB)** (crimson)...S13

Fig. S7. Absorbance changes in CA before (yellow) and after 12 min irradiation under 395 nm lamp (27 W\cm2) for Sn(PA-MPIB) (**A**), CoPC (**B**), and Sn(MPDA-PA) (**C**). All samples were tested in concentration of $1.91 \cdot 10 - 6$ [M] in ethanol. S13

Fig. S8. Cyclic voltammetry (CV) of PEI-CA (A), Sn(PA-MPIB) (B), SnPC (C), Sn(MPDA-PA) (D), and $Ru(bipy)_3$ (E). The CV was measured using 0.2M of the material in $CHCl_3$ with 0.2M $TBABF_4$. The used electrodes were gold (working electrode, -5.1 eV), Ag\AgCl (reference electrode, -4.6 eV), and platinum (counter electrode)...S14

Fig. S9. Emission spectrum of the Integration Technology Ltd.'s UV LED used for 395 nm irradiation..S15

A)

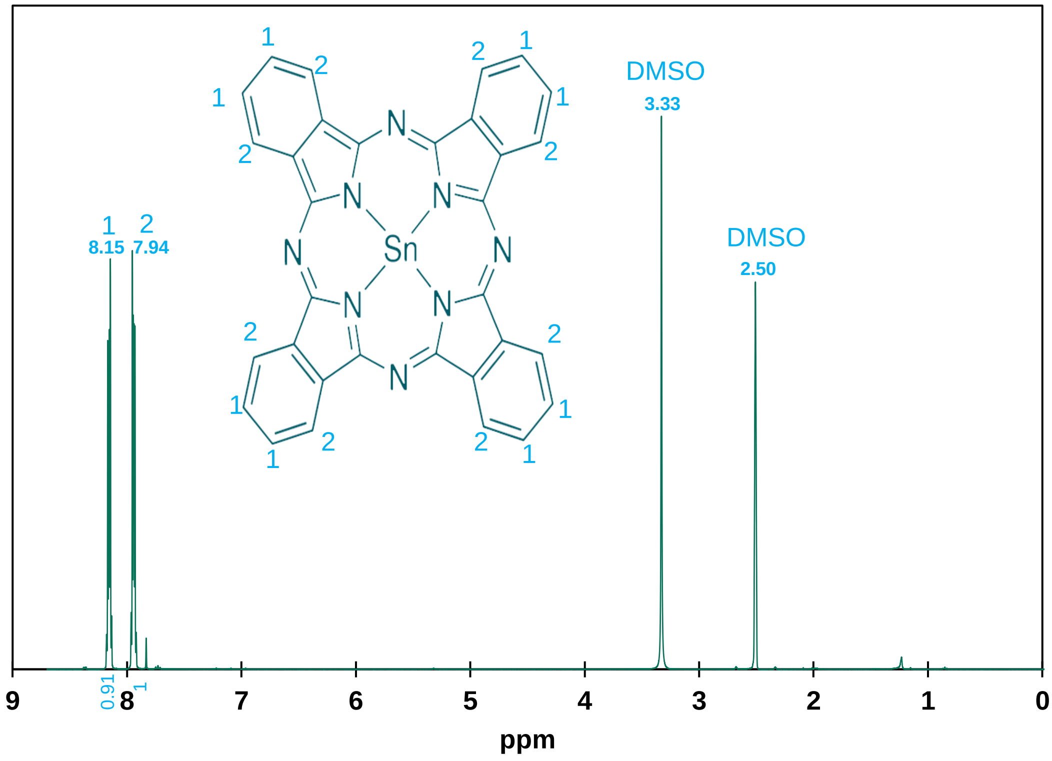

B)

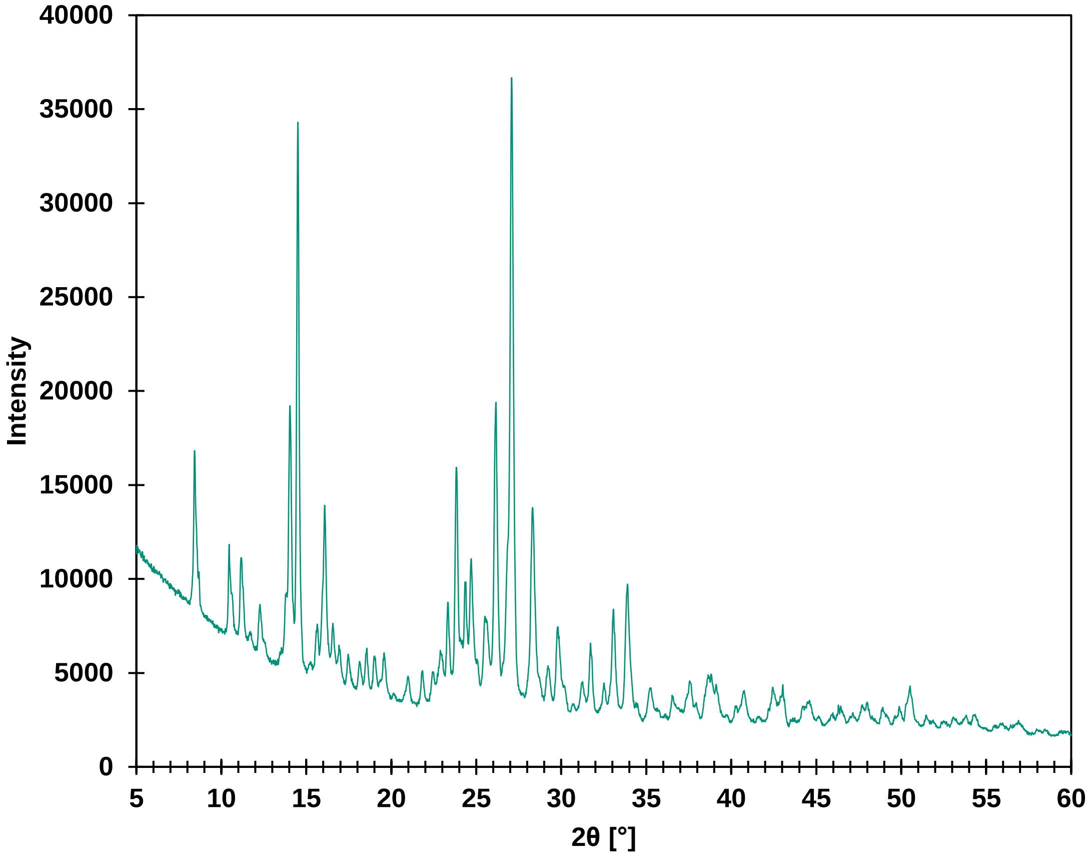

C)

2θ, deg	d, Å	Interpretation [1]
8.42	10.4887	(100) of SnPC
10.46	8.4472	(001) of $SnPCCl_2$
11.16	7.9189	(011) of $SnPCCl_2$
14.04	6.3003	(110) od SnPC
14.5	6.1015	(101) of SnPC and\or (120) of $SnPCCl_2$
16.08	5.5053	$(\bar{1}02)$ of SnPC and\or (120) of $SnPCCl_2$
16.56	5.3468	(002) of SnPC
18.56	4.7749	(300) of $SnPCCl_2$
19.6	4.5238	(220) of $SnPCCl_2$

2θ, deg	d, Å	Interpretation [1]
21	4.2253	(112) of $SnPCCl_2$
23.32	3.8099	$(\bar{1}21)$ of SnPC
23.82	3.7311	(121) of SnPC
26.16	3.4024	(310) of SnPC
27.08	3.2889	(013) of $SnPCCl_2$
28.32	3.1476	(221) of SnPC
29.22	3.0527	(203) of $SnPCCl_2$
29.8	2.9946	$(\bar{4}01)$ of SnPC
31.72	2.8175	(240) of $SnPCCl_2$
33.9	2.6412	(241) of $SnPCCl_2$

Fig. S1 . **SnPC Analysis.** ^{1}H-NMR (500 Hz) in DMSO-6D of **SnPC** (**A**), powder XRD of the complex (**B**) and an interpretation of the signals (**C**).

A)

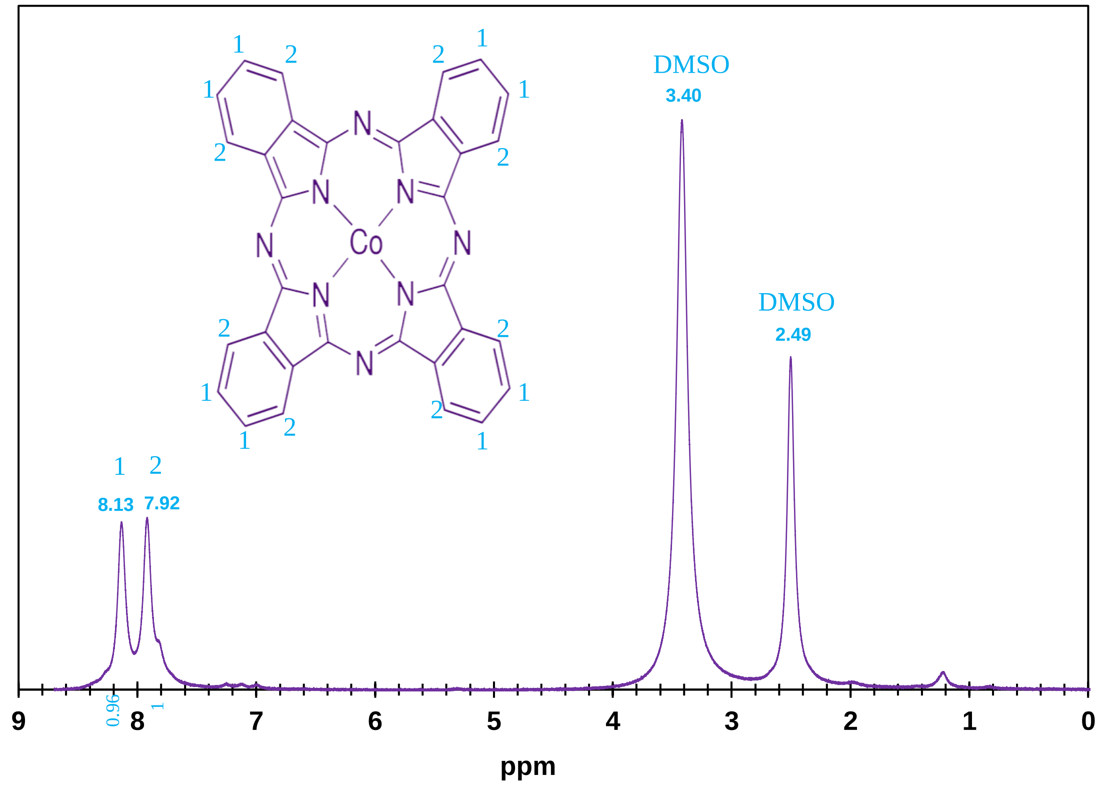

B)

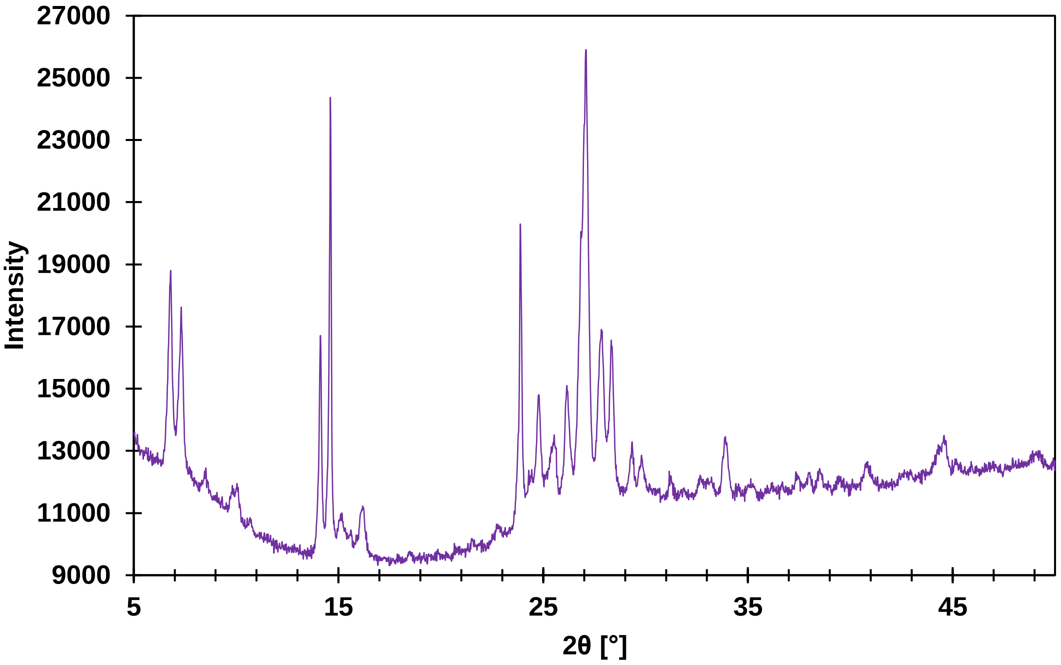

C)

2θ, deg	d, Å	Interpretation [2,3]
6.808	12.9737	α-CoPc
7.337	12.03954	α-CoPc
8.472	10.42875	CoPc Nanowire
10.036	8.8063	α-CoPc
10.707	8.25598	β-CoPc

2θ, deg	d, Å	Interpretation [2,3]
23.879	3.72334	β-CoPc
24.758	3.59321	α-CoPc
25.452	3.49676	α-CoPc
26.153	3.40454	β-CoPc
26.792	3.32482	α-CoPc

14.092	6.27966	β-CoPc	27.062	3.29224	ε-CoPc
14.601	6.06165	ε-CoPc	27.814	3.20489	α-CoPc
15.179	5.83209	CoPc Nanowire	28.322	3.14858	β-CoPc
16.172	5.47642	α-CoPc	29.329	3.04275	ε-CoPc
21.565	4.11751	β-CoPc	29.815	2.99428	CoPc Nanowire
22.769	3.90242	ε-CoPc	31.169	2.86719	β-CoPc

Fig. S2 . **CoPC Analysis.** ^{1}H-NMR (400 Hz) in DMSO-6D of **CoPC** (**A**), powder XRD of the complex (**B**) and an interpretation of the signals (**C**).

A)

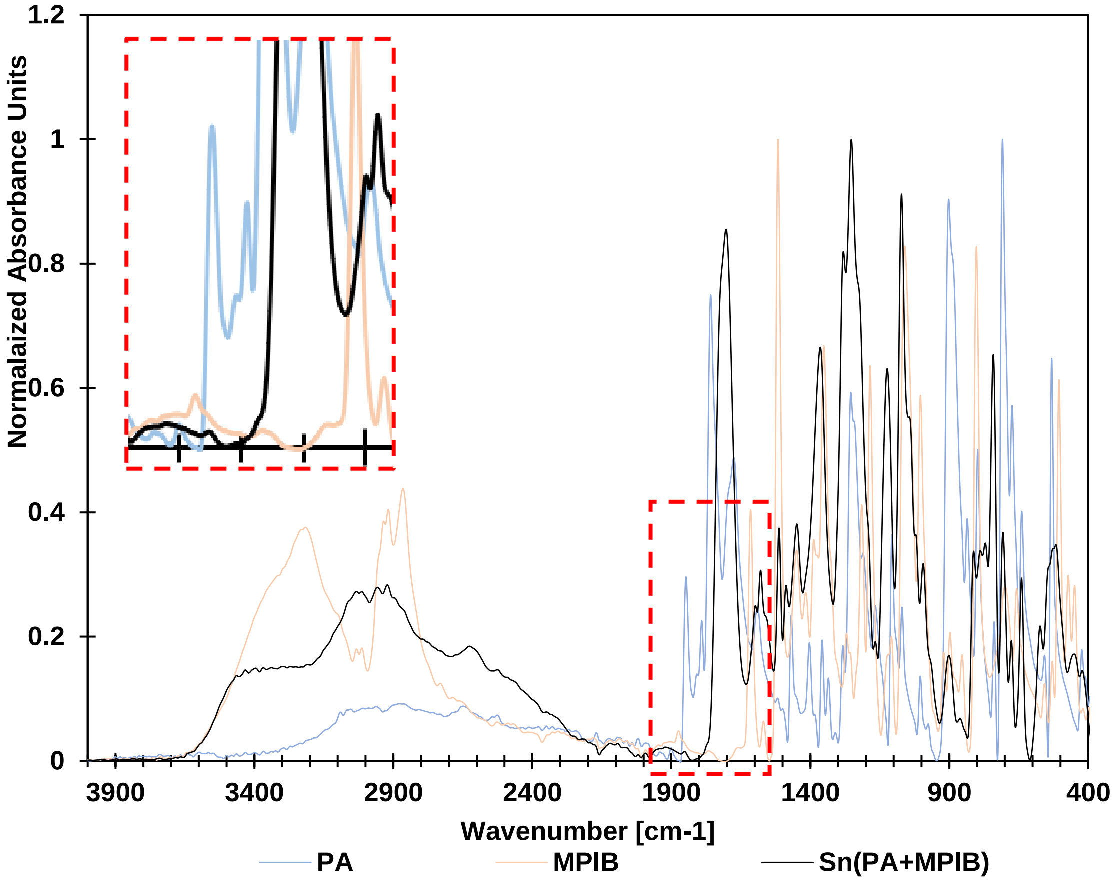

B)

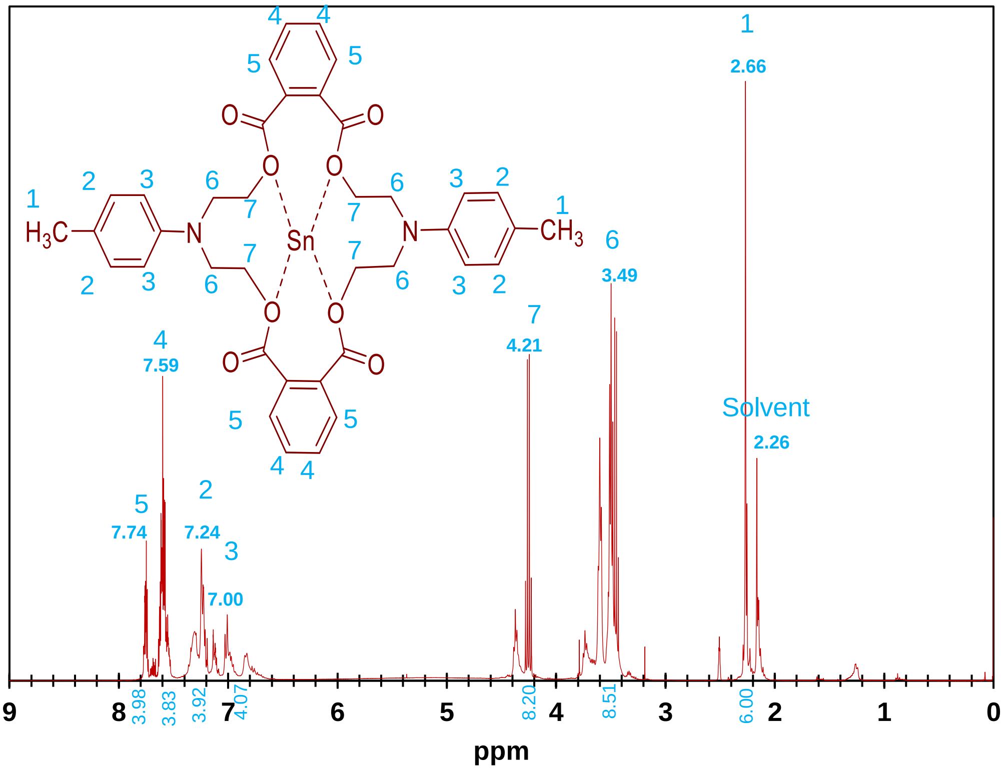

Fig. S3. **Sn(PA-MPIB) Analysis.** ATR-IR of PA (blue), MPIB (pale orange), and Sn(PA-MPIB) (black) (**A**) and ^{1}H-NMR (400 Hz) of the complex in DMSO-6D (**B**).

A)

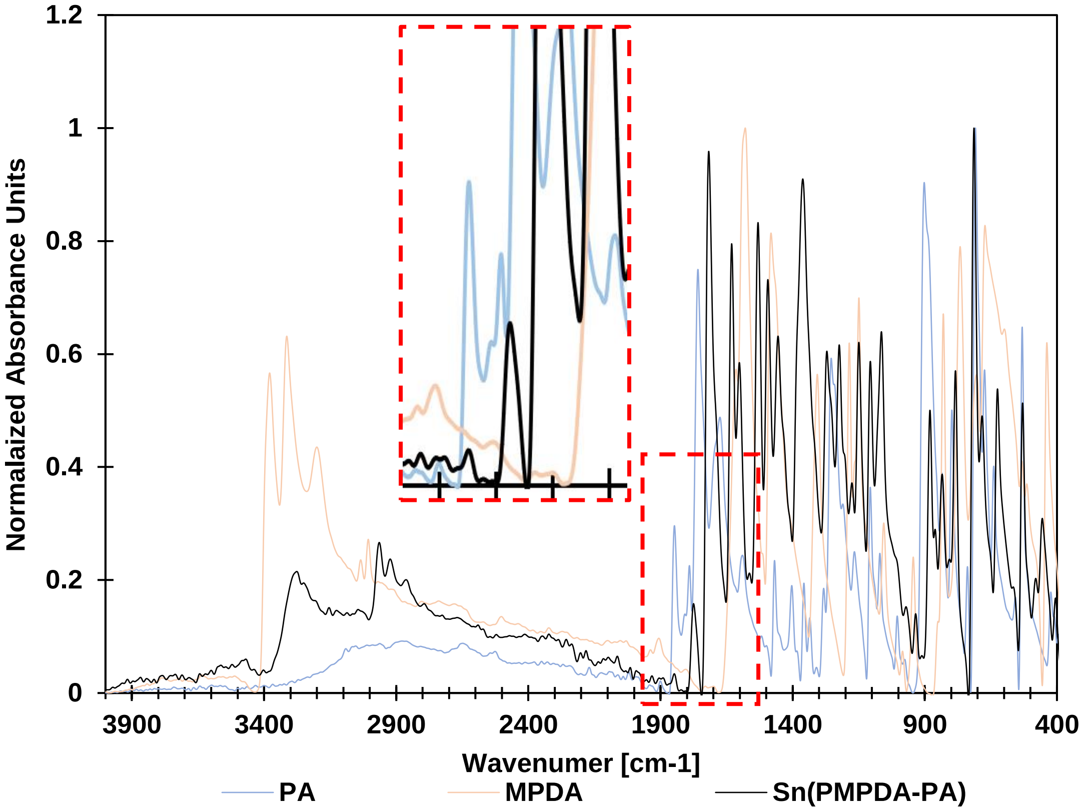
1.2
1
0.8
0.6
0.4
0.2
0
Normalaized Absorbance Units
3900
3400
2900
2400
1900
1400
900
400
Wavenumer [cm-1]
PA
MPDA
Sn(PMPDA-PA)

B)

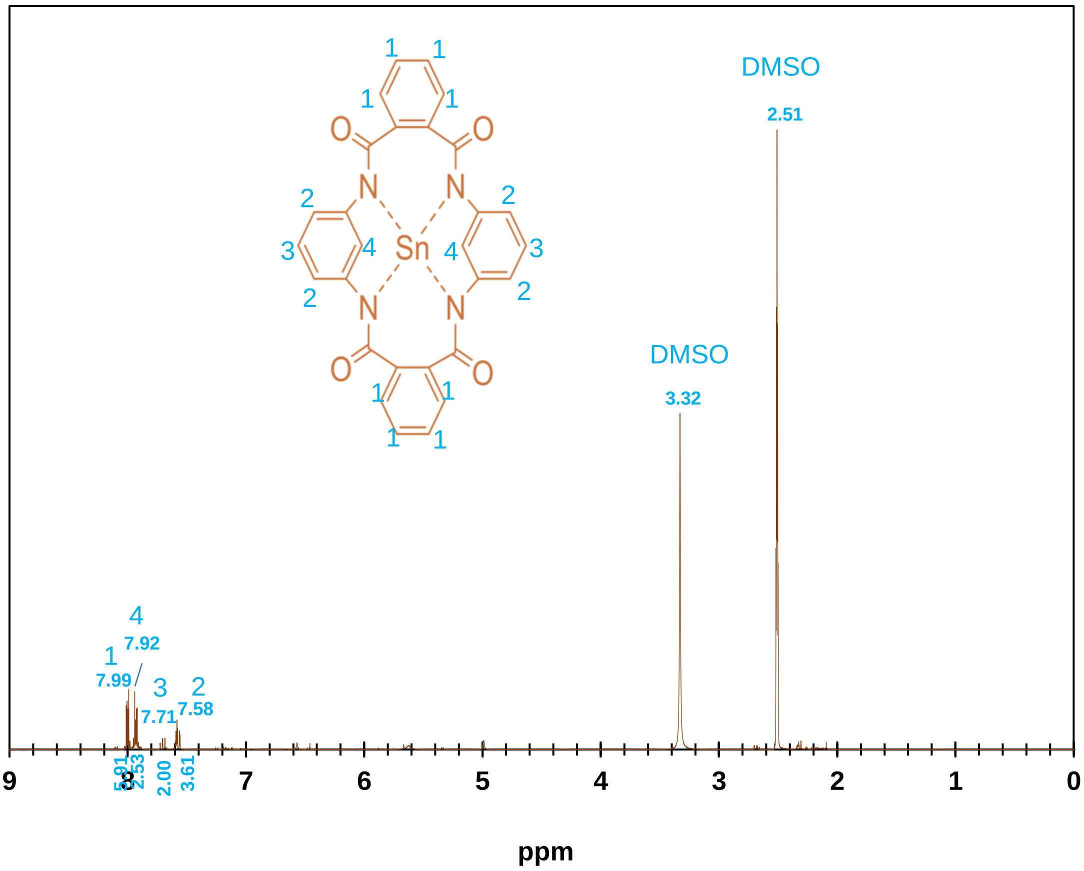
DMSO
2.51
DMSO
3.32
Sn
4
7.92
1
7.99
3
7.71
2
7.58
5.91
2.53
2.00
3.61
9
7
6
5
4
3
2
1
0
ppm

C)

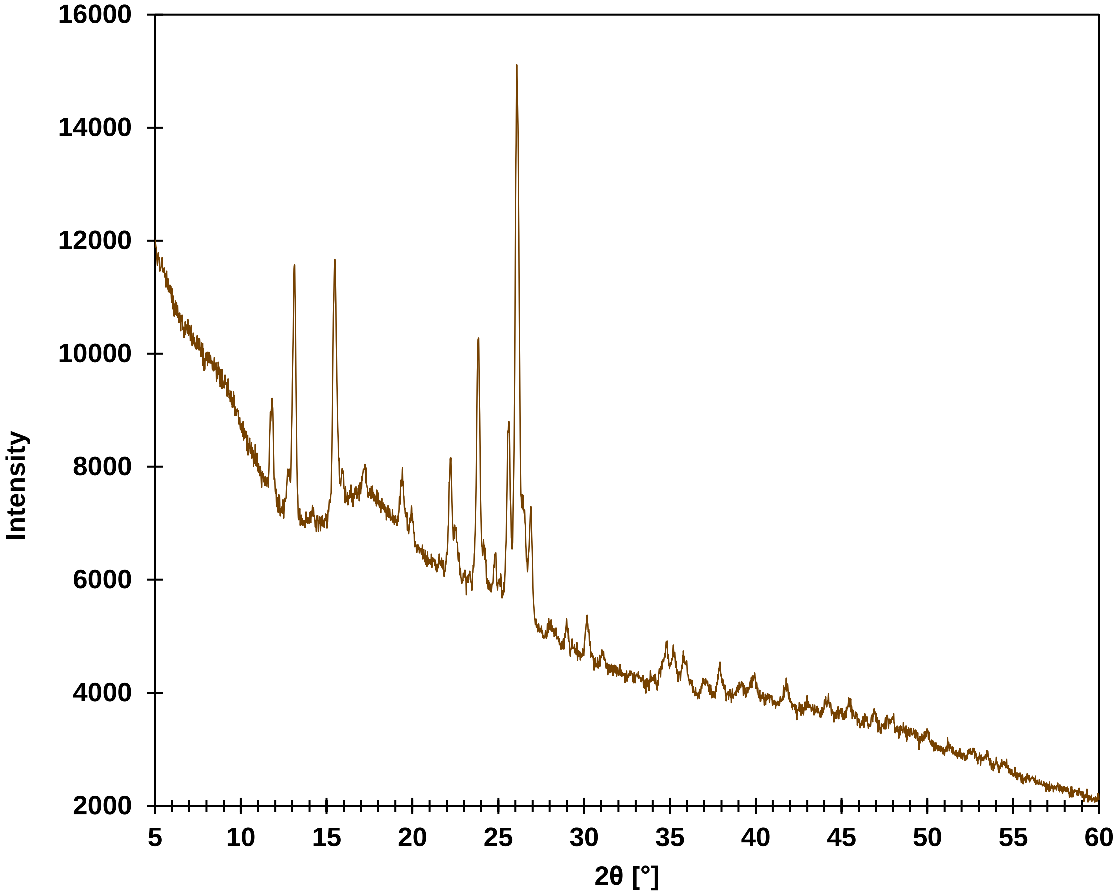

Fig. S4. **Sn(MPDA-PA) Analysis.** ATR-IR of PA (blue), MPDA (pale orange), and Sn(MPDA-PA) (black) (**A**) and ^{1}H-NMR (400 Hz, DMSO-6D) (**B**) and XRD (**C**) of the complex.

A)

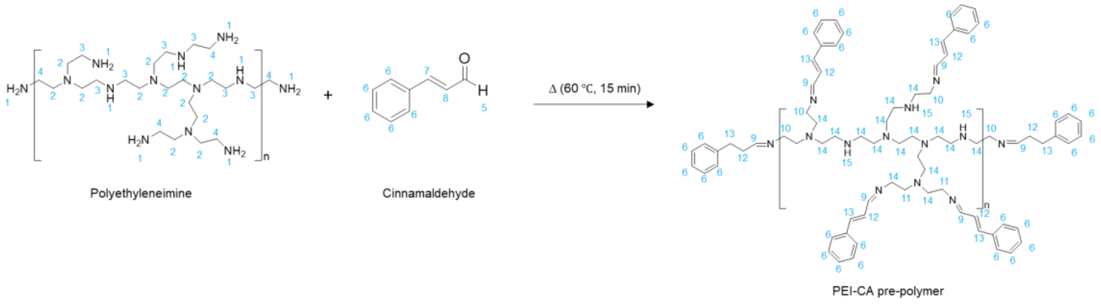

B)

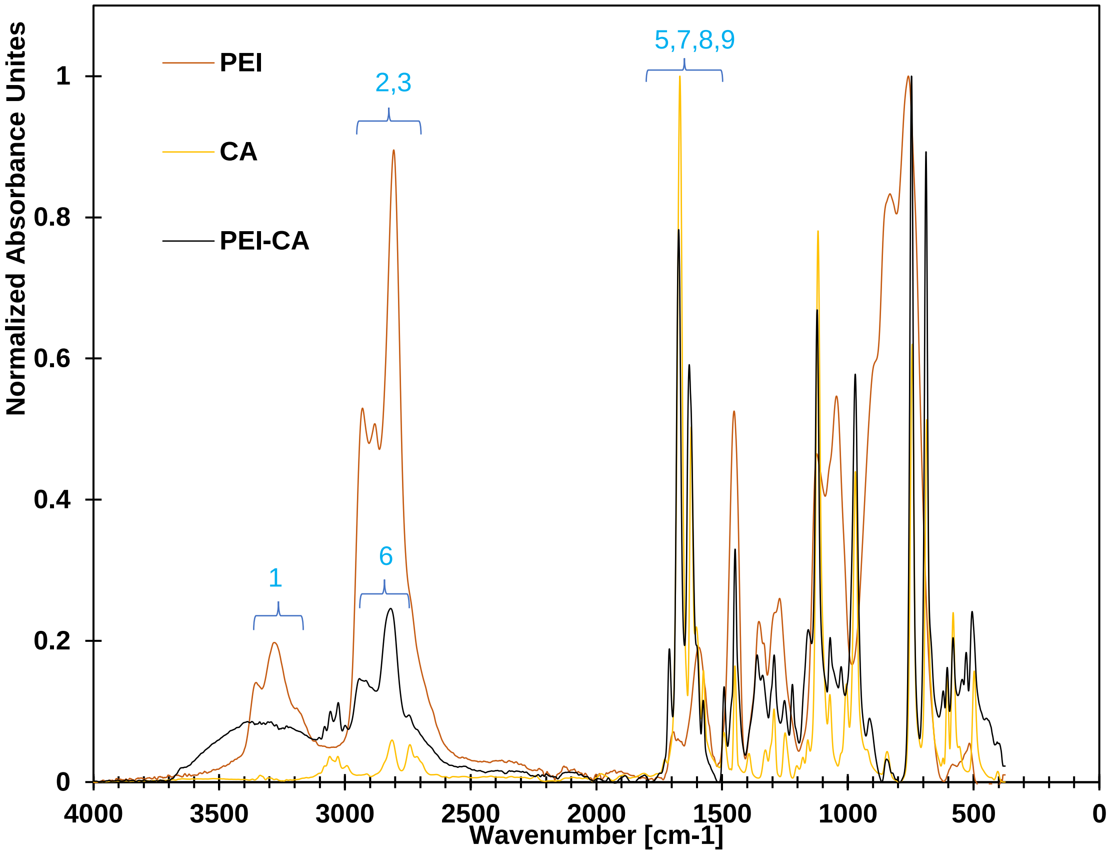

C)

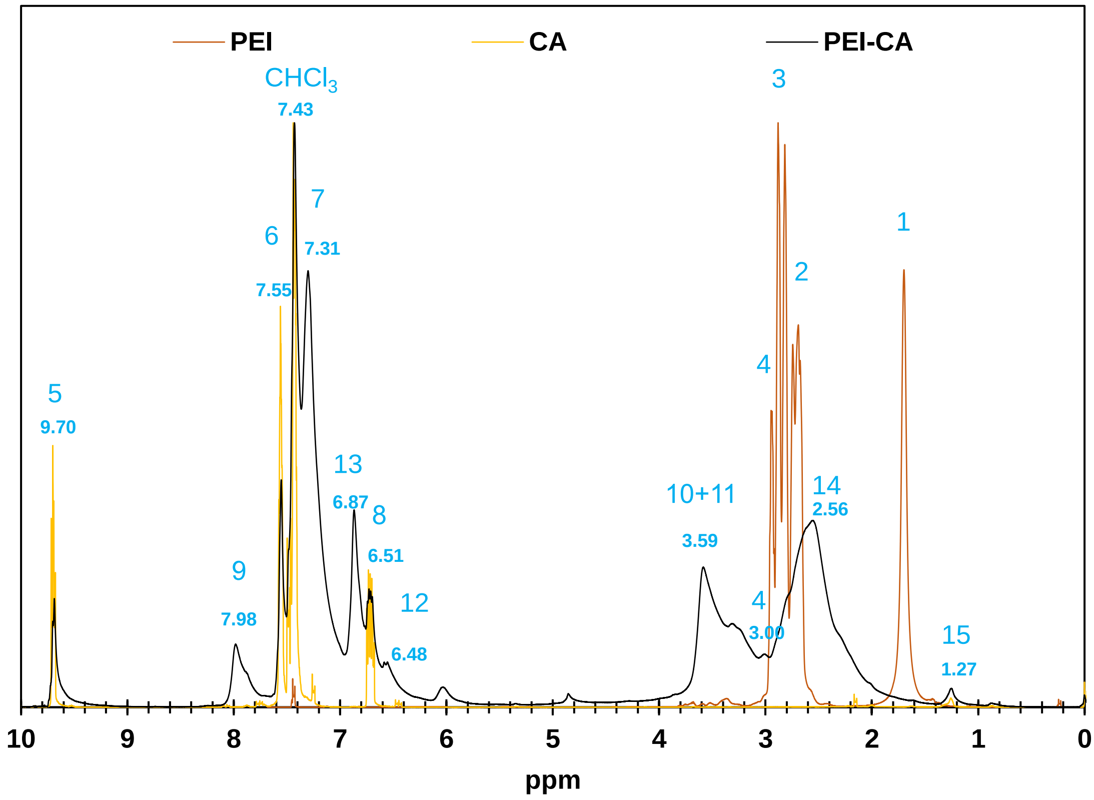

Fig. S5. **PEI-CA Analysis.** A schematic illustration of PEI-CA pre-polymer synthesis from CA and PEI (**A**) and their structure analysis by ATR-IR (**B**) and ^1H-NMR (500 Hz) in $CDCl_3$ (**C**). For both: CA (yellow), PEI (orange) and the pre-polymer **PEI-CA** (black).

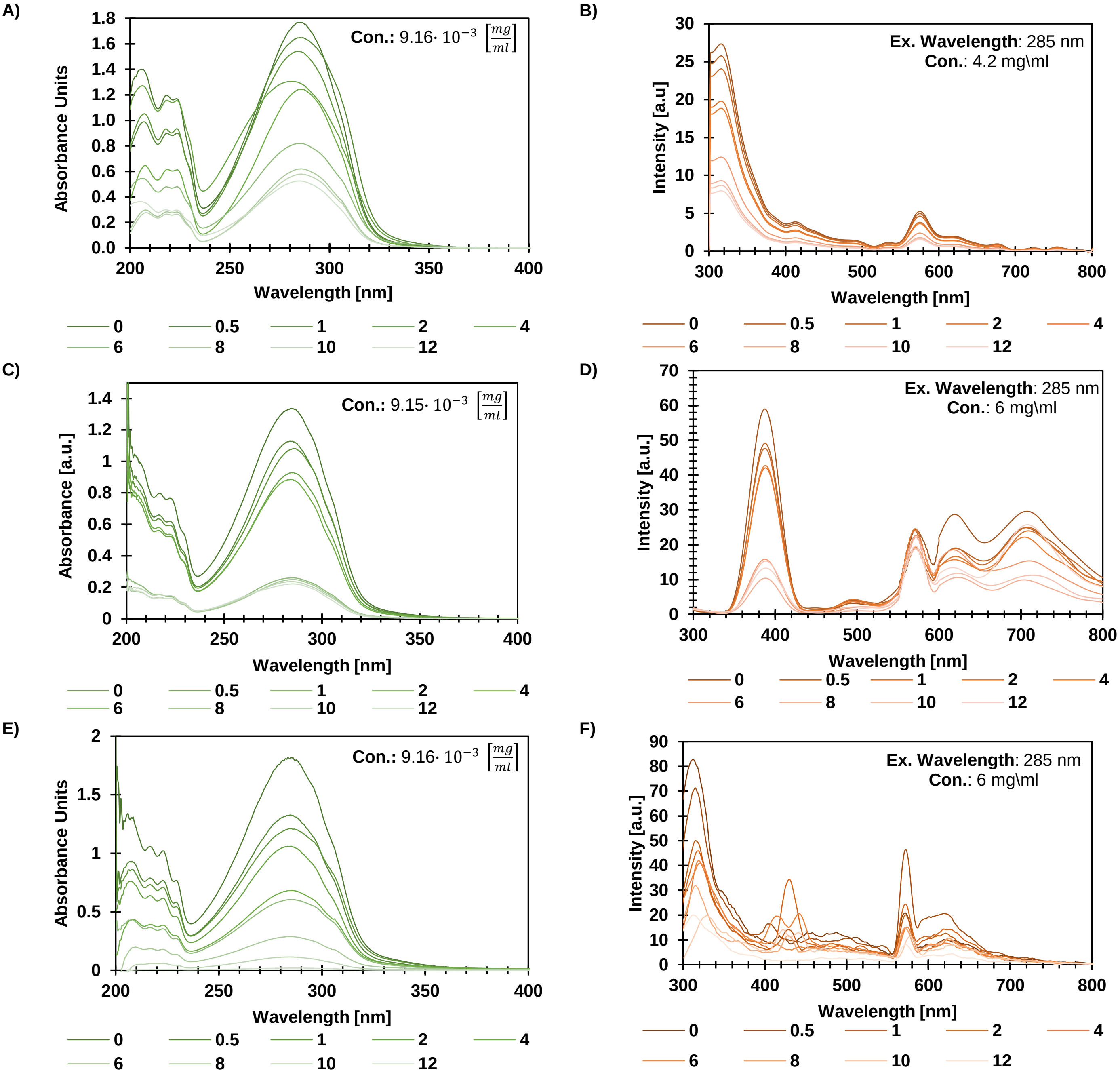
A)
Con.: 9.16· 10^-3 [mg/ml]
Absorbance Units
Wavelength [nm]
0 0.5 1 2 4 6 8 10 12
B)
Ex. Wavelength: 285 nm
Con.: 4.2 mg\ml
Intensity [a.u]
Wavelength [nm]
0 0.5 1 2 4 6 8 10 12
C)
Con.: 9.15· 10^-3 [mg/ml]
Absorbance [a.u.]
Wavelength [nm]
0 0.5 1 2 4 6 8 10 12
D)
Ex. Wavelength: 285 nm
Con.: 6 mg\ml
Intensity [a.u.]
Wavelength [nm]
0 0.5 1 2 4 6 8 10 12
E)
Con.: 9.16· 10^-3 [mg/ml]
Absorbance Units
Wavelength [nm]
0 0.5 1 2 4 6 8 10 12
F)
Ex. Wavelength: 285 nm
Con.: 6 mg\ml
Intensity [a.u.]
Wavelength [nm]
0 0.5 1 2 4 6 8 10 12

G)

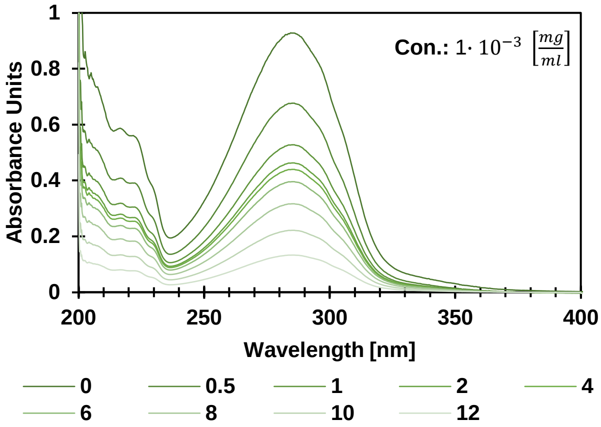
Con.: $1\cdot 10^{-3}$ $\left[\frac{mg}{ml}\right]$
Absorbance Units
Wavelength [nm]
0
0.5
1
2
4
6
8
10
12

H)

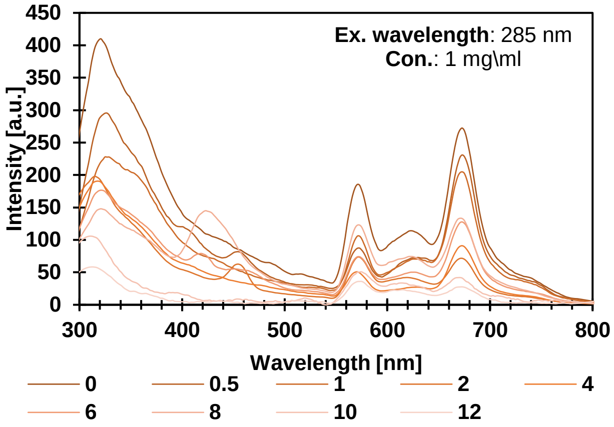
Ex. wavelength: 285 nm
Con.: 1 mg\ml
Intensity [a.u.]
Wavelength [nm]
0
0.5
1
2
4
6
8
10
12

I)

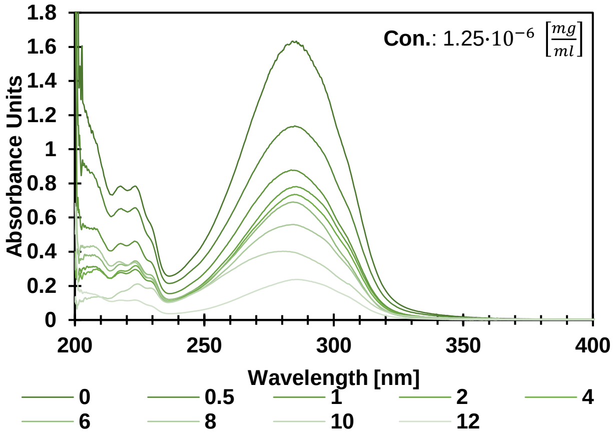
Con.: $1.25\cdot 10^{-6}$ $\left[\frac{mg}{ml}\right]$
Absorbance Units
Wavelength [nm]
0
0.5
1
2
4
6
8
10
12

J)

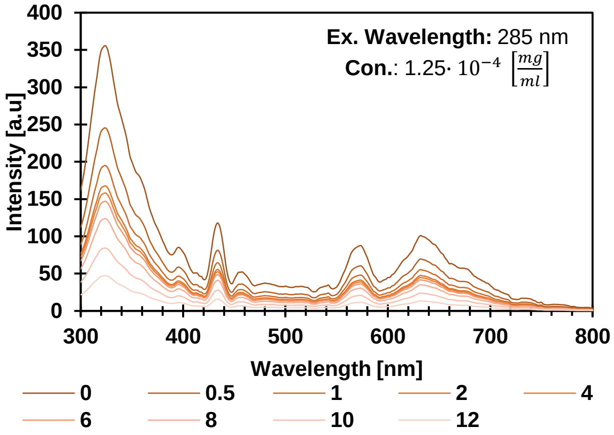
Ex. Wavelength: 285 nm
Con.: $1.25\cdot 10^{-4}$ $\left[\frac{mg}{ml}\right]$
Intensity [a.u]
Wavelength [nm]
0
0.5
1
2
4
6
8
10
12

K)

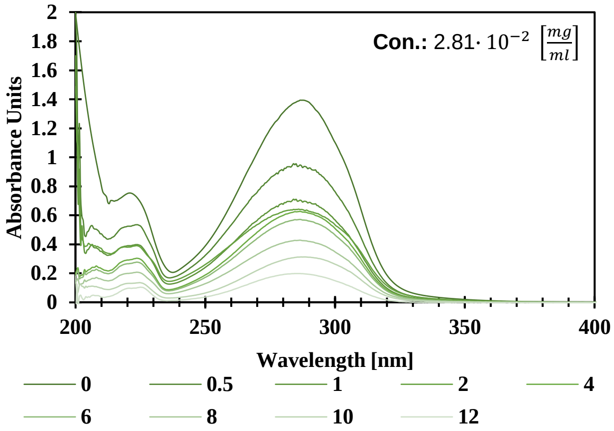
Con.: $2.81\cdot 10^{-2}$ $\left[\frac{mg}{ml}\right]$
Absorbance Units
Wavelength [nm]
0
0.5
1
2
4
6
8
10
12

L)

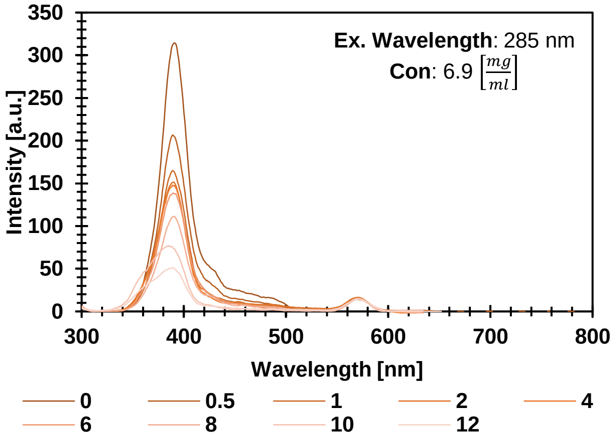
Ex. Wavelength: 285 nm
Con: 6.9 $\left[\frac{mg}{ml}\right]$
Intensity [a.u.]
Wavelength [nm]
0
0.5
1
2
4
6
8
10
12

M)

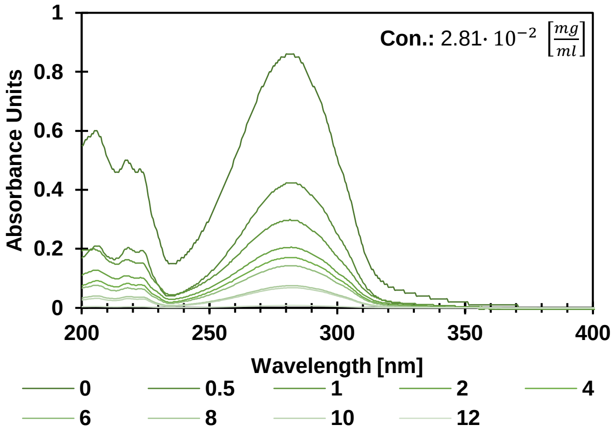
Con.: $2.81\cdot 10^{-2}$ $\left[\frac{mg}{ml}\right]$
Absorbance Units
Wavelength [nm]
0
0.5
1
2
4
6
8
10
12

N)

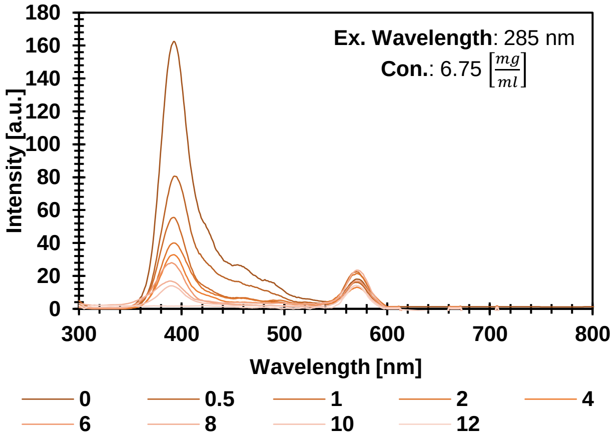
Ex. Wavelength: 285 nm
Con.: 6.75 $\left[\frac{mg}{ml}\right]$
Intensity [a.u.]
Wavelength [nm]
0
0.5
1
2
4
6
8
10
12

O)

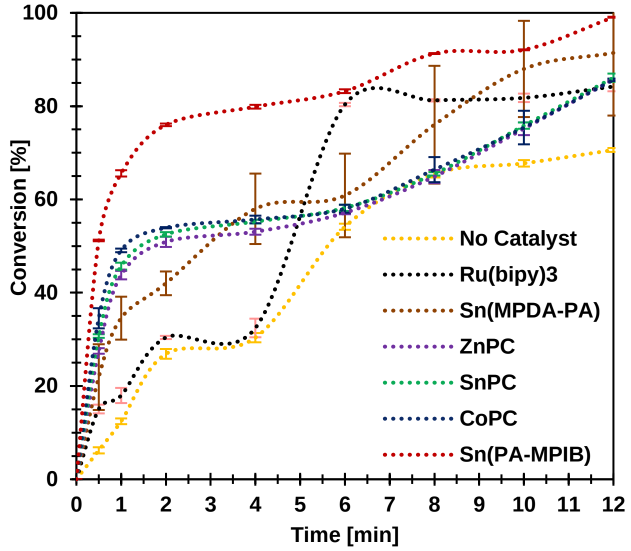

Fig. S6. PEI-CA absorbance's and fluorescence (285 nm excitation) changes after irradiation under 395 nm lamp (27 W\cm^2): without a catalyst (**A, B**), and with Ru(bipy)$_3$ (**C, D**), Sn(MPDA-PA) (**E,F**), ZnPC (**G,H**), SnPC (**I,J**), CoPC (**K,L**), and Sn(PA-MPIB) (**M,N**). The curing conversion (%) as a function of irradiation time (min) of the pre-polymer with and without the tested catalysts (**O**), where: **PEI-CA** neat (yellow), **Ru(ipy)$_3$** (black), **Sn(MPDA-PA)** (brown), **ZnPC** (purple), **SnPC** (turquoise), **CoPC** (blue), and **Sn(PA-MPIB)** (crimson).

A)

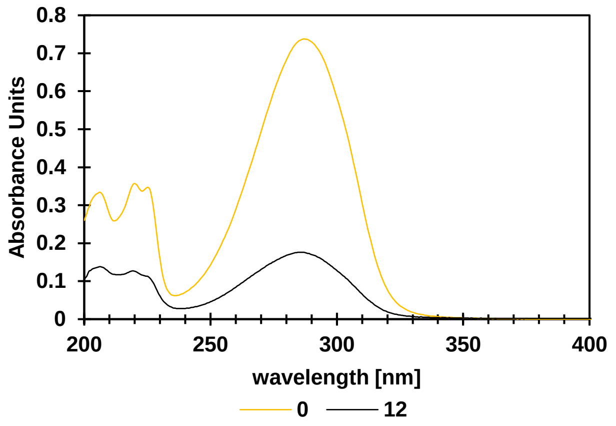

B)

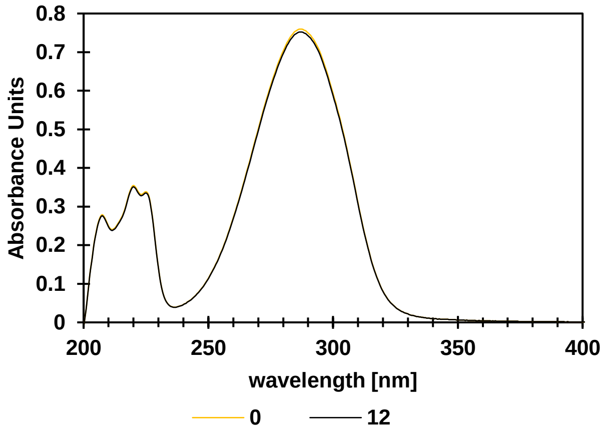

C)

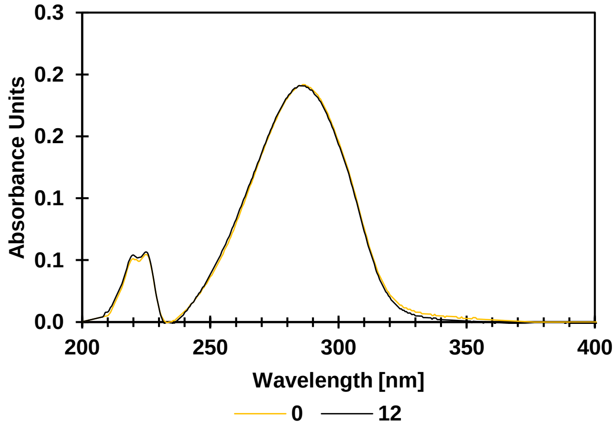

Fig. S7. Absorbance changes in CA before (yellow) and after 12 min irradiation under 395 nm lamp (27 W\cm2) for Sn(PA-MPIB) (**A**), CoPC (**B**), and Sn(MPDA-PA) (**C**). All samples were tested in concentration of $1.91 \cdot 10^{-6}$ [M] in ethanol.

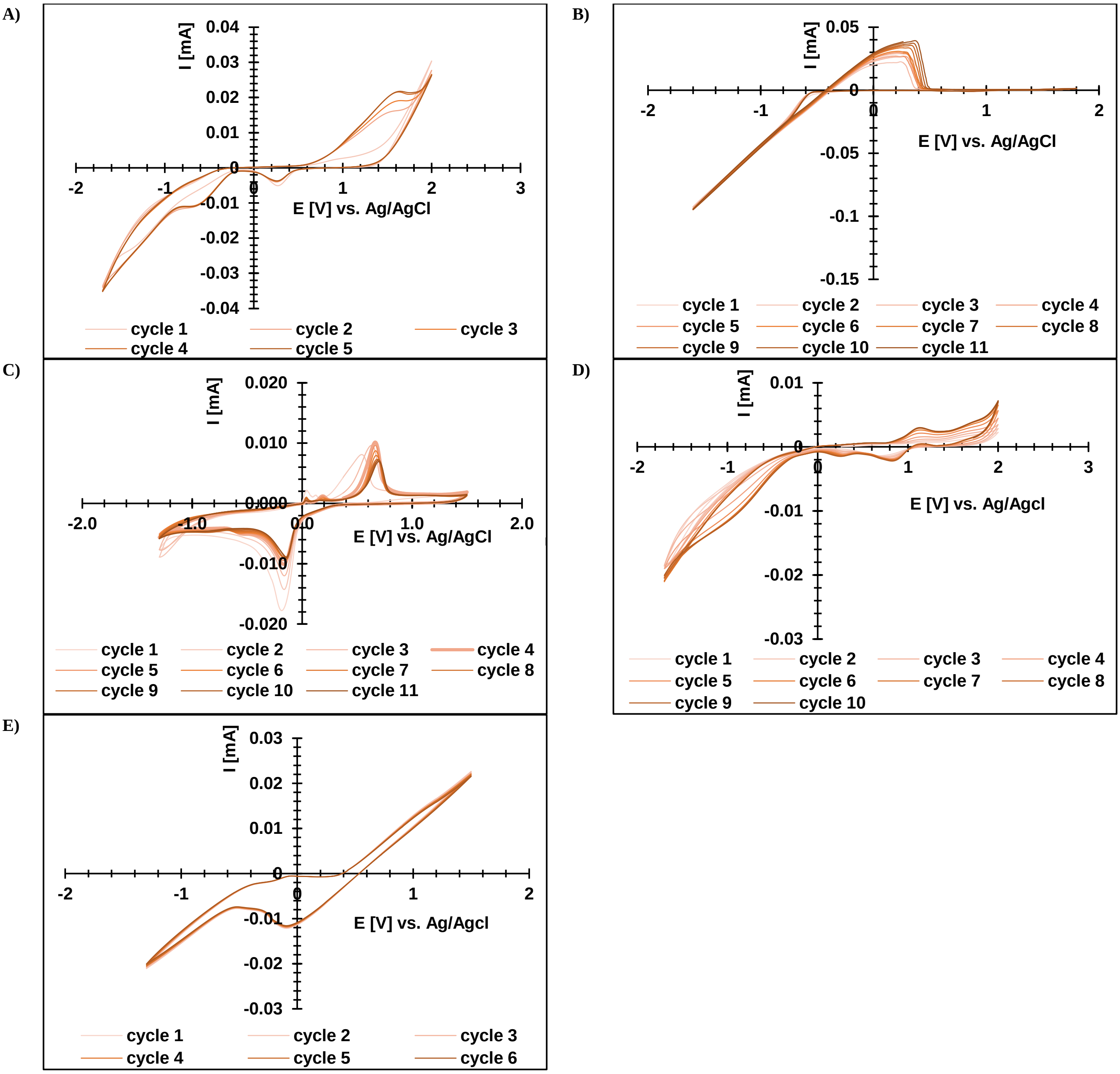

Fig. S8. Cyclic voltammetry (CV) of PEI-CA (A), Sn(PA-MPIB) (B), SnPC (C), Sn(MPDA-PA) (D), and Ru(bipy)$_3$ (E). The CV was measured using 0.2M of the material in $CHCl_3$ with 0.2M $TBABF_4$. The used electrodes were gold (working electrode, -5.1 eV), Ag\AgCl (reference electrode, -4.6 eV), and platinum (counter electrode).

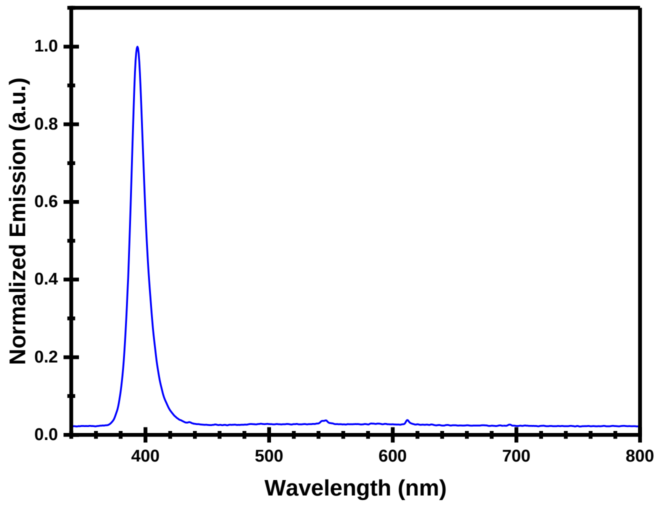

Fig. S9. Emission spectrum of the Integration Technology Ltd.'s UV LED used for 395 nm irradiation.

REFERENCES

(1) Cherian, R. C. Comparative Study of Tin Phthalocyanine and Tin Phthalocyanine Dichloride Thin Films. Induan hournal Res. 2014, 3 (9), 150–153.

(2) Enokida, T.; Hirohashi, R. Cobalt Phthalocyanine Crystal Synthesized at Low Temperature. Chem. Mater. 1991, 3 (5), 918–921. https://doi.org/10.1021/cm00017a030.

(3) Ji, X.; Zou, T.; Gong, H.; Wu, Q.; Qiao, Z.; Wu, W.; Wang, H. Cobalt Phthalocyanine Nanowires: Growth, Crystal Structure, and Optical Properties. Cryst. Res. Technol. 2016, 51 (2), 154–159. https://doi.org/10.1002/crat.201500244.
